# Impact of hospital formulary intervention on carbapenem use: a segmented time-series analysis of consumption and a propensity score-matched non-inferiority study of treatment efficacy

**DOI:** 10.1186/s40780-025-00409-6

**Published:** 2025-01-29

**Authors:** Nakaba Okamura, Ayano Katagiri, Tomoya Komori, Kei Kawanabe, Hirofumi Koike, Yukiko Sahashi, Rie Kubota

**Affiliations:** 1 Laboratory of Clinical Pharmacy Education, Research and Education Center for Clinical Pharmacy, School of Pharmacy, Kitasato University, 5-9-1, Shirokane, Minato-ku, Tokyo, 108-8641 Japan; 2 Pharmaceutical Department, Yokohama City University Hospital, 3-9. Fukuura, Kanazawa- ku, Yokohama city, Kanagawa 236-0004 Japan

**Keywords:** Pharmaceutical formulary, DRPM, Health economic evaluation, Antibiotics, Antimicrobial stewardship

## Abstract

**Background:**

Pharmaceutical formularies play a crucial role in guiding medication use by balancing clinical effectiveness and cost efficiency. Although formulary implementation has been increasing in Japan, comprehensive evaluations of its impact on both clinical and economic outcomes are limited. This study aimed to assess the effect of introducing an antimicrobial formulary at Yokohama City University Hospital on antibiotic usage and treatment outcomes in intra-abdominal infections.

**Methods:**

We conducted a segmented time-series analysis to evaluate changes in carbapenem usage, including doripenem, before and after formulary implementation in October 2018. Monthly antibiotic consumption was measured by antibiotic use density (AUD). The primary outcomes were changes in doripenem use and treatment efficacy for intra-abdominal infections. To assess treatment efficacy, we used non-inferiority analysis with propensity score matching based on age, sex, body mass index, cancer status, and baseline blood test results. The treatment outcomes were evaluated using predefined clinical indicators.

**Results:**

Following the formulary intervention, doripenem use significantly decreased from 10.8 to 4.9%, meropenem use slightly increased, and imipenem/cilastatin usage remained stable. Overall, carbapenem use significantly decreased during the study period. Treatment effectiveness for intra-abdominal infections remained non-inferior, with a higher proportion of patients classified as having an “effective” response post-intervention (86.6% vs. 79.4% pre-intervention). The confidence interval confirmed the non-inferiority margin, indicating no clinically significant reduction in treatment effectiveness following the formulary introduction.

**Conclusions:**

The introduction of an antibiotic formulary at Yokohama City University Hospital effectively reduced the use of doripenem without compromising the effectiveness of treatment of intra-abdominal infections. These findings suggest that formulary management can be a valuable strategy for optimizing antibiotic use while maintaining clinical outcomes and contributing to improved antimicrobial stewardship in healthcare settings. Further research is warranted to explore the broader implications of formulary implementation in Japanese healthcare practices.

**Supplementary Information:**

The online version contains supplementary material available at 10.1186/s40780-025-00409-6.

## Background

The pharmaceutical formulary is a critical tool in modern healthcare designed to optimize patient care by facilitating the selection of cost-effective and clinically appropriate medications. Formularies play a central role in guiding therapeutic decisions, ensuring that medication use aligns with the latest clinical evidence and best practices while also controlling healthcare costs. According to the American Society of Health-System Pharmacists (ASHP) guidelines, a pharmaceutical formulary is “a continually updated list of available medications and related information, representing the clinical judgment resulting from a review of the clinical evidence of physicians, pharmacists, and other clinicians in the diagnosis, prophylaxis, or treatment of disease and promotion of health.” [1].

The importance of pharmaceutical formularies has been widely recognized globally, particularly for their role in improving healthcare outcomes and optimizing resource utilization. Formularies have been linked to enhanced prescription efficiency, reduced drug expenditure, and improved patient outcomes. However, several studies have reported potential challenges, including the risk of limiting therapeutic options and negative effects on clinical outcomes [2]. The findings from previous studies underscore the need to balance cost-effectiveness with clinical efficacy in formulary implementation [3].

In Japan, the adoption of pharmaceutical formularies has experienced significant growth in recent years. Several hospitals and healthcare institutions have documented their experience with formulary implementation, reporting positive effects on drug utilization and cost management [4, 5]. Despite these advancements, there is a lack of comprehensive studies evaluating both the clinical and economic outcomes of formulary introduction.

Given these gaps in the literature, we aimed to conduct a comprehensive evaluation of the hospital antimicrobial formulary from the perspective of changes in usage and treatment effectiveness at Yokohama City University Hospital. Our study assessed both the clinical effectiveness and economic efficiency of the formulary, providing a balanced analysis of its impact on healthcare outcomes. By addressing both of these aspects, we hope to contribute valuable insights to the broader discussion on formulary management and its implications for healthcare in Japan.

## Methods

We conducted a segmented time-series analysis to evaluate changes in carbapenem usage following the implementation of a hospital pharmaceutical formulary. Additionally, a non-inferiority test with propensity score matching was performed to assess the changes in treatment efficacy. The study period spanned from September 1, 2016, to October 31, 2020. The effects of the formulary introduction were assessed by comparing usage before (September 1, 2016, to September 30, 2018) and after (October 1, 2018, to October 31, 2020) the intervention, including changes in the target areas of usage. We examined the impact on the effectiveness of infectious disease treatment from two perspectives.

### Hospital formulary for carbapenem antibiotics at Yokohama city university hospital

In October 2018, we formulated the standard use of carbapenem antibiotics in our hospital and administered them throughout the hospital. At the time this formulary was implemented, 5 carbapenem antibiotics were available on the Japanese market: meropenem (MEPM), biapenem (BIPM), imipenem/cilastatin (IPM/CS), panipenem/betamiprone (PAPM/BP), and doripenem (DRPM). Our hospital has used all 5 types. Before the formulary intervention, the selection of carbapenem antibiotics was not restricted, and the choice among carbapenems (e.g., MEPM, DRPM, and IPM/CS) was made at the discretion of the prescribing physician. At our institution, all carbapenem usage requires submission of a usage report to the Infection Control Department for monitoring purposes.

Based on evidence regarding therapeutic efficacy and safety, drug prices, usage history, and other factors, 3 types of carbapenem antibiotics are recommended as standards (Appendix 1). The first recommendation was MEPM, which is highly recommended by various guidelines in the field of infectious diseases, and has a relatively low cost due to its status as a generic drug. Despite limited evidence and its higher cost, DRPM was chosen as the second most recommended drug. Additionally, IPM/CS was selected as the second recommended drug due to its extensive clinical experience and pharmacokinetic data in pediatric and neonatal populations in Japan [6–8], was selected as the second recommended drug. This formulation was approved by the Pharmaceutical Affairs Committee of Yokohama City University Hospital and was issued throughout the hospital. After the system was made public, a list of standard recommended drugs popped up when entering a prescription in the electronic medical record.

### Changes in antimicrobial use

The subjects were patients who received injectable antibiotics (MEPM, DRPM, and IPM/CS) during hospitalization at Yokohama City University Hospital during the survey period. There were no exclusions based on the patient characteristics. The monthly usage of formulary-eligible injectable antibiotics (DRPM, IPM/CS, MEPM) administered to target cases was calculated from the electronic medical records of Yokohama City University Hospital. The total amount of carbapenem used was calculated as a reference value.

Given the Japanese pharmaceutical pricing system, where drug prices are periodically reduced, and the potential for total drug costs to be influenced by the allocation of multiple product specifications (e.g., 0.5 g/vial and 1 g/vial), economic evaluations based on drug prices may be influenced by systemic factors. To avoid this potential bias and provide a more practical and accurate assessment, we evaluated economic efficiency based on antibiotic usage volume.

The amount used was calculated using the ATC/DDD system published by WHO [9] and the AUD was calculated. The following formula was used to calculate the AUD: The World Health Organization’s ATC index version 2022 was used for DDD.

$$\begin{aligned}AUD&=\frac{\begin{aligned}&Monthly\:usage\:amont\:of\:Targeted\:Antibiotics\,(g)\end{aligned}}{\begin{aligned}&DDD(g/person\:per\:day)\times\:total\:number\:of\:inpatient\:days(person\:per\:day)\end{aligned}}\times\:1000\end{aligned}$$

DDD: Defined Daily Dose.

The evaluation item was whether there was a decrease in the use of DRPM, the second most recommended drug, due to the introduction of the hospital formulary. The calculated AUD was analyzed using a generalized linear model to perform a split time-series analysis before and after the intervention [10]. The only factors included in the generalized linear model were time course and formulary intervention factors. Model selection was performed by comparison with Akaike’s information criterion (AIC) [11, 12]. A generalized linear model was employed with a gamma distribution and an inverse link function to model the relationship between DRPM usage and formulary intervention. For autocorrelation evaluation, residuals, autocorrelation, and partial autocorrelation were checked.

### Treatment effect for intra-abdominal infections

The subjects were hospitalized patients with intra-abdominal infections who received injectable antibiotics for 3 or more days during the study period. Target diseases included peritonitis (including pelvic inflammatory disease), intra-abdominal abscess, and hepatic/biliary tract infections (e.g., cholecystitis, cholangitis, and liver abscess). From the electronic medical records of the Yokohama City University Hospital, we identified patients who met these criteria. Patients with intra-abdominal infections were selected as the target population based on the usage patterns of carbapenem at our institution. Prior to the formulary intervention, DRPM was frequently used in gastrointestinal surgery, while IPM/CS had limited usage. Given the frequent use of DRPM and abundant evidence supporting its efficacy in intra-abdominal infections, this domain was deemed appropriate for evaluating changes in usage patterns and clinical outcomes associated with the formulary intervention.

Patient selection was further refined based on the exclusion criteria listed in Appendix 2. This exclusion criterion is based on the definitions outlined in *Clinical Evaluation Methods for Antibacterial Drugs for Intra-Abdominal Infections* by the Ministry of Health, Labour, and Welfare in Japan, which specifies clinical evaluation standards for antibacterial agents used in intra-abdominal infections [13]. Additionally, cases in which antibiotic treatment was not intended for the management of intra-abdominal infections were excluded.

Treatment effectiveness was assessed using the following indicators: (1) body temperature < 37 °C, (2) white blood cell count within the normal range, (3) C-reactive protein (CRP) level below 3.0 mg/dL, (4) improvement in abdominal findings, and (5) resolution of abnormal imaging findings. The criteria for evaluating treatment effectiveness were based on the evaluation items used in phase III clinical trials of antibacterial treatments for intra-abdominal infections conducted in Japan [14]. These criteria were adjusted to include items that could be observed in medical records. If 4 of these 5 indicators were met at 14 days after the initiation of antibiotic therapy or at the final treatment day (whichever occurred earlier), the response was considered “excellent”. A response meeting 2–3 indicators was classified as “effective”, while responses meeting only ≤ 1 were deemed “ineffective”. If ≥ 3 were not measured, the outcome was classified as “indeterminate”. Cases classified as “indeterminate” were excluded from the analysis to ensure the reliable assessment of treatment outcomes.

The proportion of patients categorized as “effective” or better was compared before and after formulary introduction using a non-inferiority test [15]. Propensity score matching (PSM) was applied to control for baseline covariates, including age at admission, sex, BMI, cancer status, and baseline blood test results (WBC, RBC, albumin, AST, ALT, ALP, and CRP) [16, 17]. Propensity scores were calculated using logistic regression, and covariates with a standardized mean difference (SMD) of ≥ 0.15 were adjusted in the final analysis. We employed 1:1 nearest-neighbor matching without replacement, using a caliper width of 0.1 of the standard deviation of the logit of the propensity score.

The primary outcome of the non-inferiority test was the proportion of patients categorized as “effective” or better. To ensure that the formulary change did not lead to a clinically significant reduction in effectiveness, a non-inferiority margin of 10% points was selected based on clinical relevance and previous studies [18–20]. We used the likelihood method to evaluate non-inferiority with a one-sided significance level of 0.05. Non-inferiority was established if the lower bound of the 95% confidence interval (CI) for the difference in effectiveness exceeded a pre-specified margin.

Additionally, if physician comments regarding the adverse effects of antibiotic use were present in patient records, they were documented and counted.

### Statistical analysis

All statistical processing and figure creation were performed using R (ver. 4.3.2) [21]. The following libraries were used: tidyverse, tableone, ggplot2, MatchIt, mice, cobalt, and gridExtra [22–28]. P values of < 0.05 were considered to indicate statistical significance.

## Results

### Modifications in antimicrobial utilization

During the observation period at our institution, 3,443 patients received carbapenem antibiotics, with 1,835 patients starting treatment before the intervention and 1,608 patients starting treatment after the intervention (Table 1). Among these, 719 patients required repeated courses of carbapenem antibiotics.

**Table 1:** Carbapenem usage before and after intervention

	Before		After	
**n**	**1835**		**1608**	
Sex = female, n (%)	694	(37.8)	674	(41.9)
Age, mean (SD)	63.0	(19.9)	62.3	(21.3)
**Medication**,** n (%)**				
**MEPM**	**1577**	**(85.9)**	**1451**	**(90.2)**
Internal Medicine	580	(31.6)	558	(34.7)
Surgery	750	(40.9)	558	(34.7)
Gastrointestinal Surgery	530	(28.9)	353	(22.0)
Others	247	(13.5)	334	(20.8)
**DRPM**	**198**	**(10.8)**	**79**	**(4.9)**
Internal Medicine	27	(1.5)	14	(0.9)
Surgery	103	(5.6)	20	(1.2)
Gastrointestinal Surgery	102	(5.5)	19	(1.2)
Others	68	(3.7)	45	(2.8)
**IPM/CS**	**60**	**(3.3)**	**78**	**(4.9)**
Internal Medicine	14	(0.8)	18	(1.1)
Surgery	34	(1.9)	40	(2.5)
Gastrointestinal Surgery	4	(0.2)	24	(1.5)
Others	12	(0.7)	20	(1.2)
Treatment duration days, mean (SD)	8.4	(7.6)	7.3	(6.5)

MEPM, meropenem; DRPM, doripenem; IPM/CS, imipenem/cilastatin

The usage patterns of carbapenem antibiotics shifted significantly following the formulary intervention. MEPM usage increased from 85.9 to 90.2%, while DRPM usage decreased markedly from 10.8 to 4.9%. IPM/CS usage remained relatively stable, showing a slight increase from 3.3 to 4.9%. Notably, carbapenem antibiotics were primarily utilized in surgical departments before the intervention, accounting for 48.3% of total usage, compared to 33.8% in internal medicine departments. The data indicate that DRPM was primarily used in the surgery department before the intervention, particularly in gastrointestinal surgery, which accounted for 103 of 198 cases (52.0%). Following the intervention, DRPM usage in gastrointestinal surgery significantly decreased to 19 cases. After the implementation of the formulary, the proportion of carbapenem use in surgical departments decreased significantly to 38.4%, while usage in internal medicine departments increased slightly to 36.9%.

No significant differences were observed in patient demographics, including sex composition and age distribution, between the pre- and post-intervention periods. However, the average duration of carbapenem administration decreased from 8.4 days (SD: 7.6) to 7.3 days (SD: 6.5). Among surgical specialties, gastroenterological surgery demonstrated the most notable reduction in carbapenem usage, with its proportion decreasing from 28.2% before the intervention to 16.7% after the intervention.

**Fig. 1:**
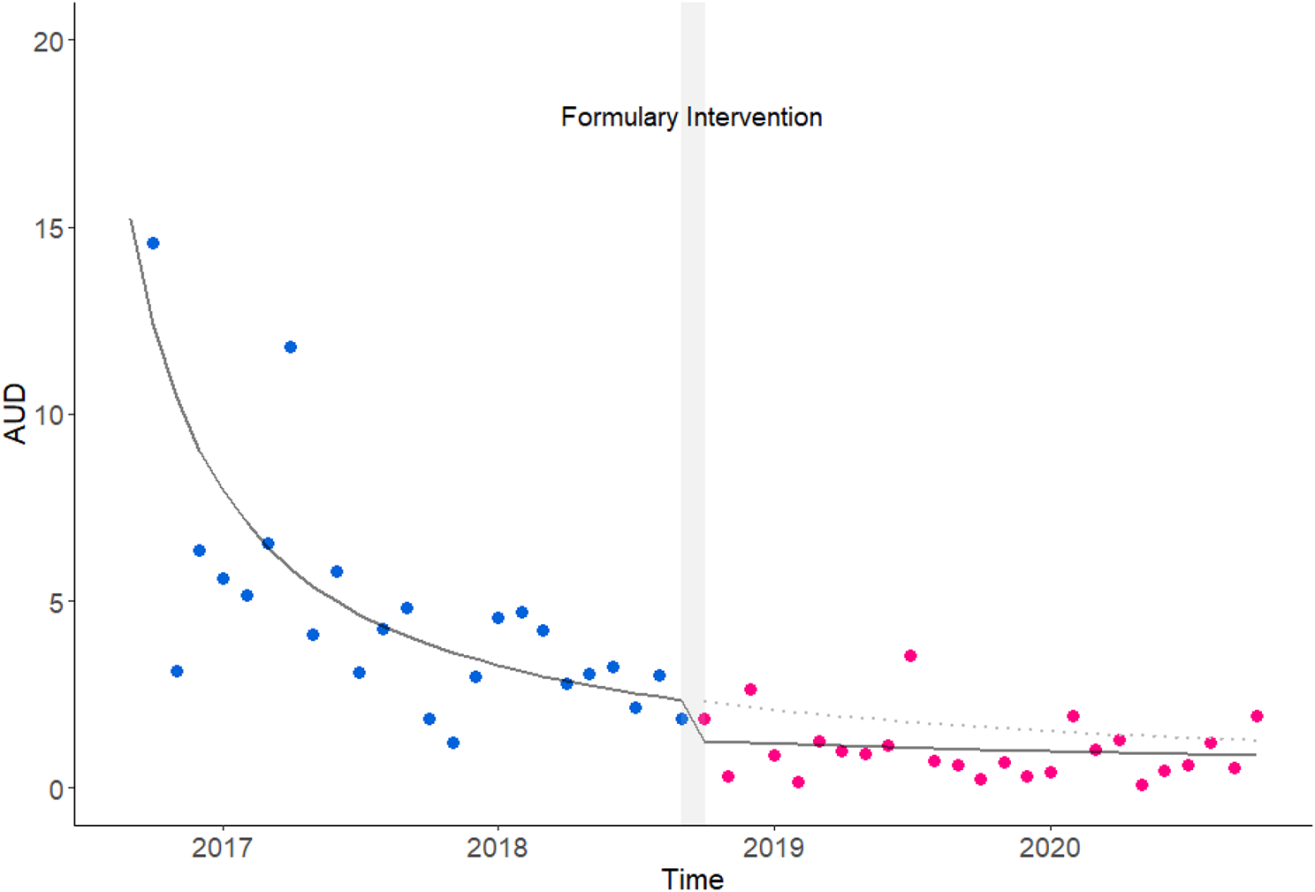
Changes in DRPM AUDs following hospital formulary intervention

A generalized linear model was used to analyze the monthly AUDs for individual agents and carbapenems. AIC values were 165.07 for DRPM, 296.13 for MEPM, and 111.32 for IPM/CS. Temporal trends in monthly AUD, derived from the model, showed that DRPM had a significant downward trend over time (estimate = -0.015, 95% CI: -0.0083 to -0.2199, *p* < 0.001), with further reduction associated with formulary intervention (Estimate = -0.364, 95% CI: -0.0514 to -0.6895, *p* < 0.05) (Table 2; Fig. 1). No significant temporal or intervention-related changes were observed for MEPM or IPM/CS. The overall AUD for carbapenems decreased significantly over the study period (Estimate = -0.00055, 95% CI: -0.00026 to -0.00085, *p* < 0.001).

**Table 2:** Effects of hospital formulary intervention on usage of carbapenems

Medications	Factors	Estimates	95%CI
MEPM	Time	<0.001	-0.000057–0.000773
	Intervention	0.035	-0.008652–0.015632
DRPM	Time	0.015	0.008299–0.021986
	Intervention	0.365	0.051363–0.689466
IPM/CS	Time	-0.003	-0.026506–0.020826
	Intervention	-0.165	-0.835623–0.509664
Carbapenems	Time	<0.001	0.000256–0.000848
	Intervention	0.002	-0.007286–0.010327

### Outcomes of intra-abdominal infection management

**Fig. 2:**
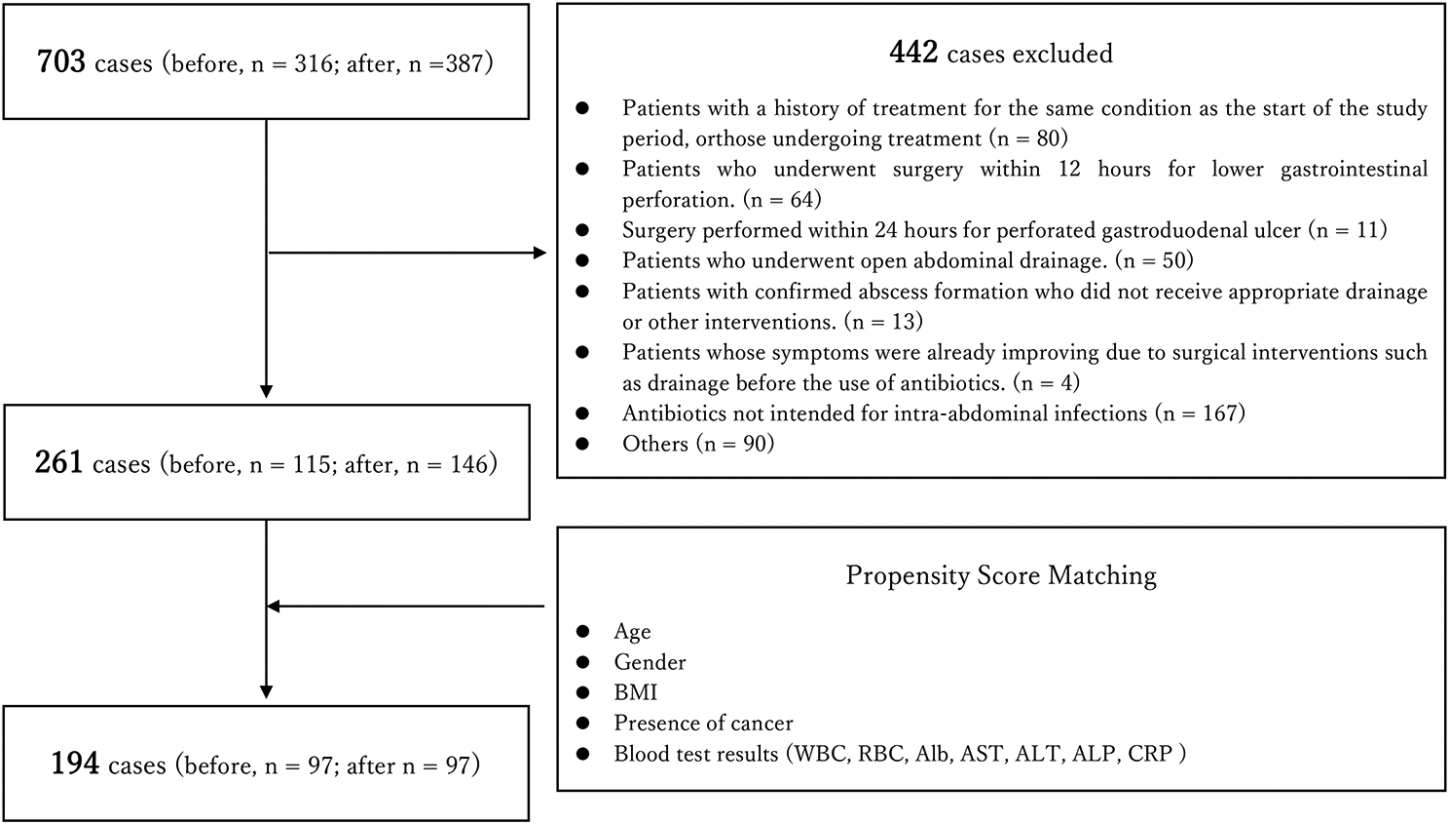
Changes in DRPM AUDs following hospital formulary intervention

From our institution’s electronic medical records, and in accordance with the predetermined selection criteria, a total of 703 cases were identified. Of these, 442 cases were excluded based on the exclusion criteria (Fig. 2). After exclusion, 261 cases remained (before intervention, *n* = 115; after intervention, *n* = 146) (Fig. 2). Of the 18 variables collected from the electronic medical records, age, sex, BMI, cancer presence, WBC, RBC, serum albumin levels, AST, ALT, ALP, and CRP were included as covariates for propensity score matching (Table 3).

**Table 3:** Baseline characteristics in the original and matched samples

	Original					Matched				
	Before		After		SMD	Before		After		SMD
Confounders	**115**		**146**			**97**		**97**		
Age, mean (SD)	69.05	(11.44)	69.86	(13.16)	0.065	69.80	(11.24)	70.29	(11.02)	0.044
Female, n (%)	35	(30.4)	52	(35.6)	0.110	31	(32.0)	29	(29.9)	0.045
BMI, mean (SD)	21.21	(3.51)	21.93	(3.25)	0.214	21.47	(3.66)	21.37	(2.95)	0.030
Cancer = TRUE, n (%)	88	(76.5)	91	(62.3)	0.020	73	(75.3)	70	(72.2)	0.070
WBC, mean (SD)	10942.61	(7340.12)	9900.00	(4732.63)	0.169	9741.24	(4871.28)	9836.08	(4712.49)	0.020
RBC, mean (SD)	386.36	(73.57)	380.65	(68.26)	0.080	381.08	(69.71)	383.70	(66.81)	0.038
ALB, mean (SD)	3.25	(0.57)	3.38	(0.64)	0.214	3.28	(0.57)	3.33	(0.61)	0.098
T-Bil, mean (SD)	2.08	(2.38)	2.31	(2.88)	0.088	2.13	(2.53)	2.23	(2.44)	0.041
AST, mean (SD)	123.79	(177.89)	178.50	(410.06)	0.173	128.85	(184.36)	145.54	(318.37)	0.064
ALT, mean (SD)	97.39	(117.97)	144.54	(244.91)	0.245	103.24	(124.09)	111.49	(150.15)	0.060
ALP, mean (SD)	799.23	(695.59)	724.94	(634.41)	0.112	779.91	(654.17)	753.36	(635.46)	0.041
LDH, mean (SD)	267.51	(154.25)	294.34	(279.35)	0.119	266.51	(155.67)	292.24	(260.42)	0.120
γGTP, mean (SD)	321.40	(338.52)	311.88	(330.70)	0.028	313.35	(321.41)	310.53	(339.29)	0.009
SCr, mean (SD)	0.88	(0.47)	0.86	(0.35)	0.059	0.88	(0.49)	0.89	(0.39)	0.004
eGFR, mean (SD)	70.70	(23.24)	70.03	(23.60)	0.028	70.24	(21.95)	69.76	(25.12)	0.020
CRP, mean (SD)	8.87	(7.55)	6.52	(7.23)	0.317	7.54	(6.93)	7.54	(7.95)	< 0.001
Cases using DRPM	**9**		**1**			**7**		**1**		

**Fig. 3:**
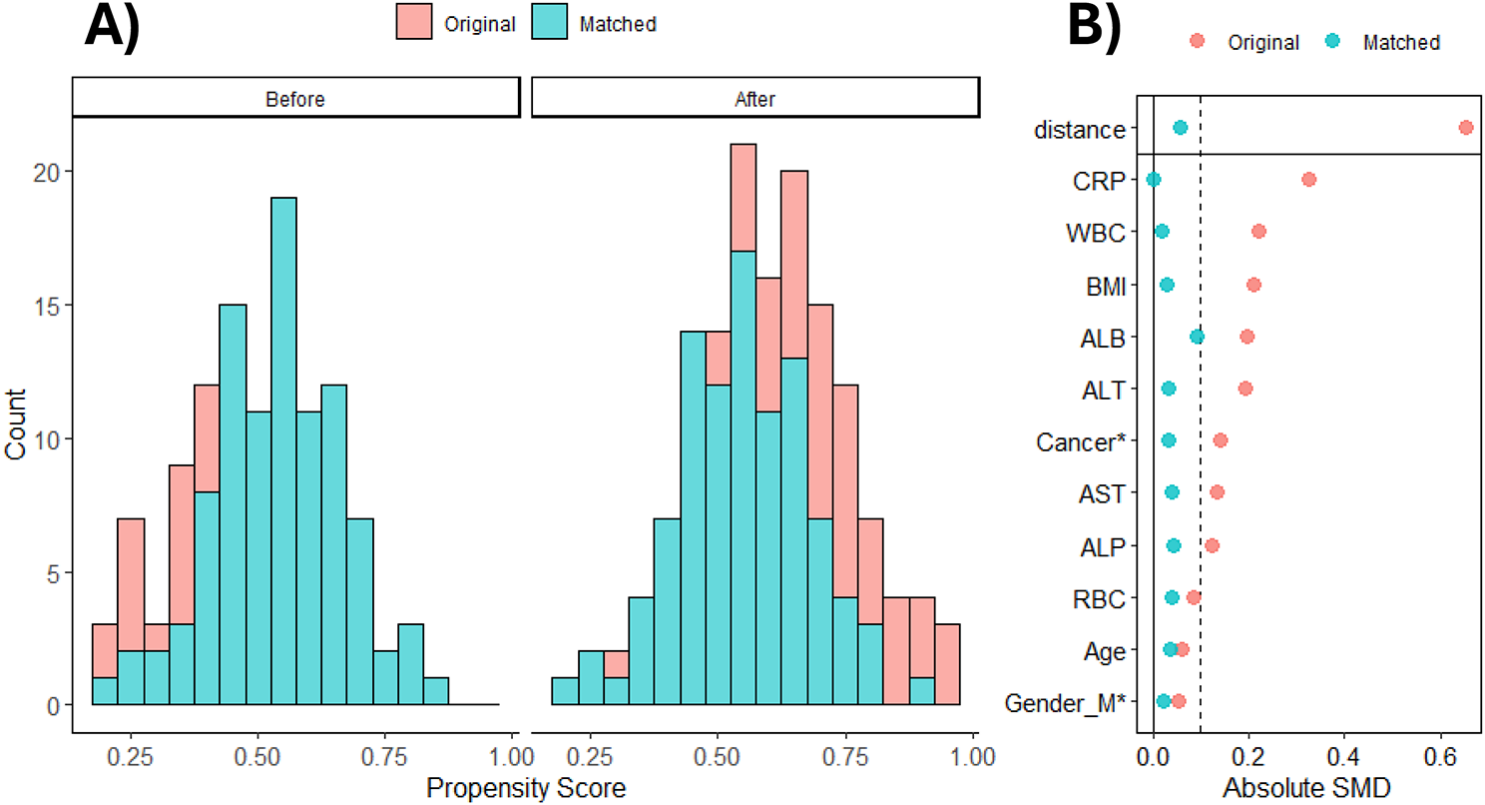
**A**) Distribution of propensity scores between original data and matched data, **B**) Covariate balance Original, original data; Matched, matched data

Figure 3 illustrates the distribution of propensity scores before and after matching as well as the standardized mean differences (SMD) for key covariates. As shown in Fig. 3A, the propensity score distribution demonstrated a significant improvement in the overlap between the intervention and control groups after matching. The matched sample (blue) shows a much tighter and more comparable distribution than that of the original sample (red). Figure 3B displays the absolute SMD for each covariate before and after matching. After matching, most covariates achieved SMD < 0.1, indicating a well-balanced comparison between the groups. For instance, variables such as age, BMI, and CRP level showed substantial improvements in balance. While some covariates such as LDH and albumin retained higher SMDs, the overall balance was markedly improved, as indicated by the closer alignment of points with the dotted line at 0.1.

The mean treatment duration was 11.07 days (SD: 7.43) in the pre-intervention group and 10.51 days (SD: 4.88) in the post-intervention group(Table 4). The rate of antibiotic switching decreased from 31 (32.0%) in the pre-intervention group to 14 (14.4%) in the post-intervention group. Adverse effects were observed in 5 cases (5.2%; 1 case each of liver dysfunction, renal dysfunction, rash, diarrhea, and decreased blood cell count) in the pre-intervention group and 3 cases (3.1%; 2 cases of liver dysfunction and 1 case of rash) in the post-intervention group.

**Table 4:** Clinical outcomes and antibiotic use before and after the intervention in the matched cohorts

	Before		After	
	*n* = 97		*n* = 97	
Outcome = effective, n (%)	77	(79.4)	84	(86.6)
Excellent	12	(12.4)	13	(13.4)
Effective	65	(67.0)	71	(73.2)
Treatment duration(days), mean (SD)	11.07	(7.43)	10.51	(4.88)
Switching Antibiotics = TRUE, n (%)	31	(32.0)	14	(14.4)
Adverse effect = TRUE, n (%)	5	(5.2)	3	(3.1)
Antibiotics, n (%)				
Anti-MRSA	6	(6.2)	3	(3.1)
Cephalosporine (2nd)	8	(8.2)	8	(8.2)
Cephalosporine (3rd /4th)	3	(3.1)	1	(1.0)
Carbapenem	32	(33.0)	11	(12.4)
MEPM	24	(24.7)	9	(9.3)
DRPM	7	(7.2)	1	(1.0)
IPM/CS	1	(1.0)	1	(1.0)
βl-Penicillin	77	(79.4)	47	(48.5)
SBT/ABPC	29	(29.9)	10	(10.3)
TAZ/PIPC	48	(49.5)	37	(38.1)
βl-Cephalosporine	11	(11.3)	46	(47.4)
SBT/CPZ	11	(11.3)	46	(47.4)
Others	4	(4.1)	1	(1.0)

MRSA, methicillin-resistant *Staphylococcus aureus*; cephalosporine (2nd), second-generation cephalosporines; cephalosporine(3rd /4th), third-generation and fourth-generation cephalosporins; βl-Penicillin, penicillins combined with β-lactamase inhibitors; βl-Cephalosporin, cephalosporin combined with β-lactamase inhibitor

Regarding the specific antibiotics used, the administration of carbapenems significantly decreased from 32 cases (33.0%) in the pre-intervention group to 11 cases (12.4%) in the post-intervention group. In particular, the use of meropenem (MEPM) decreased from 24 cases (24.7%) to 9 cases (9.3%), and the use of doripenem (DRPM) decreased from 7 cases (7.2%) to 1 case (1.0%). Conversely, the use of β-lactamase inhibitor-combined cephalosporins increased from 11 cases (11.3%) in the pre-intervention group to 46 cases (47.4%) in the post-intervention group, whereas the use of β-lactamase inhibitor-combined penicillins decreased from 77 (79.4%) to 47 (48.5%).

Within the pre-intervention group (97 cases), DRPM was administered in 7 cases (7.2%), compared to 1 case (1.0%) in the post-intervention group. Therapeutic outcomes were effective in 77 patients (79.4%) in the pre-intervention group and 84 patients (86.6%) in the post-intervention group. The difference in effectiveness between the two groups was statistically significant, with a 95% confidence interval of -0.045480 to 0.17180 (*p* < 0.01), supporting the non-inferiority of the post-intervention treatment outcomes.

## Discussion

Our study demonstrated that implementation of the hospital antibiotic formulary significantly reduced the use of DRPM, which was relegated to a second-line antibiotic option in our institution. Furthermore, the treatment outcomes for intra-abdominal infections, where DRPM was primarily used, showed non-inferiority after the intervention.

At Yokohama City University Hospital, the introduction of the hospital formulary for carbapenem antibiotics significantly reduced the use of DRPM, whereas no notable changes were observed in the use of MEPM or IPM/CS. The proportion of DRPM prescriptions among all carbapenem antibiotics decreased, suggesting an interventional effect of the hospital formulary.

However, it is important that the overall prescription volume of carbapenem antibiotics has been decreasing over time, even before the implementation of the formulary, which is considered to be a result of antimicrobial stewardship initiatives within the hospital. 　This finding aligns with the antibiotic stewardship program (ASP) in Japan. A national plan to combat antimicrobial resistance (AMR) was established in 2013 to promote the appropriate use of antibiotics. In our research period, there have been numerous reports on the reduction in the use of carbapenem antibiotics through interventions by ASPs or antimicrobial stewardship teams (ASTs) [29–31]. In our facility, the AST monitors the prescription of specific antimicrobials, including carbapenem antibiotics, piperacillin/tazobactam (PIPC/TAZ), injectable fluoroquinolones, and anti-MRSA agents. Additionally, AST supervises antimicrobial prescriptions exceeding 14 days, cases with positive blood cultures, and cases in the intensive care unit [32].

Before the intervention, DRPM was a commonly used option in the Department of Gastroenterological Surgery for treating intra-abdominal infections, such as cholangitis, cholecystitis, pancreatitis, pancreatic fistula, peritonitis, and intra-abdominal abscesses. Several studies have documented the effectiveness of DRPM in these areas [33]. After the intervention, DRPM use in these areas decreased, enabling a comparison of treatment outcomes to confirm non-inferiority. Our findings showed that the therapeutic effectiveness of antibiotics in these areas remained non-inferior before and after formulary intervention, implying that the change in antibiotic choice did not lead to inferior treatment outcomes.

These results are consistent with other reports from Japan, where formulary interventions have been shown to alter prescription volumes and trends [34, 35], often focusing on balancing cost-effectiveness and promoting the appropriate use of antibiotics [36, 37]. The essential impact of formulary interventions should be evaluated by balancing economic assessments with the non-inferiority of treatment outcomes, which distinguishes this study from previous research. Physicians prioritize “treatment effectiveness and safety” over “economic efficiency” when selecting antibiotics [38], and our findings contribute to enhancing the persuasiveness of standard interventions such as drug formularies by reinforcing this balance.

This study had some limitations. First, the change in antibiotic usage occurred during a period when carbapenem usage had already decreased owing to enhanced antimicrobial stewardship activities at our institution. This temporal trend may have overshadowed the specific impact of the formulary intervention, potentially leading to an underestimation of its effects. Second, the study population was limited to cases of intra-abdominal infections without surgical interventions in accordance with international guidelines that recommend carbapenems for moderate to severe cases. However, only approximately 10% of our study population required DRPM, possibly limiting the generalizability of our findings to the broader population of intra-abdominal infections. Future studies with larger populations are necessary to evaluate the broader impact of formulary interventions. Additionally, the overall proportion of carbapenem prescriptions differed between the pre- and post-intervention periods. This suggests that it may have been essential to either limit the analysis to patients who used carbapenems or to perform stratified analyses. In the future, we believe that to effectively evaluate the impact of formulary interventions, study designs should consider not only the target disease, but also the specific medications used by the population under evaluation.

The economic evaluation of pharmaceuticals should not solely focus on cost reduction. Instead, it should encompass a comprehensive assessment that balances treatment effectiveness with cost. We hope that formulary evaluations in Japan will increasingly adopt a bidirectional approach, ensuring that both economic considerations and patient outcomes are addressed adequately.

## Electronic supplementary material

Below is the link to the electronic supplementary material.

Supplementary Material 1

## Data Availability

No datasets were generated or analysed during the current study.

## Abbreviations

AIC: Akaike information criterion
ALP: Alkaline Phosphatase
ALT: Alanine Aminotransferase
ASHP: The American Society of Health-System Pharmacists
ASP: Antimicrobial stewardship program
AST: Aspartate Aminotransferase
AST: Antimicrobial stewardship team
ATC: The Anatomical Therapeutic Chemical classification
AUD: Antibiotic Utilization Density
BIPM: Biapenem
BMI: Body mass index
CI(95%CI): Confidence interval(95% confidence interval)
CRP: C-reactive protein
DDD: Defined Daily Dose
DRPM: Doripenem
IPM/CS: Imipenem/cilastatin
LDH: Lactate Dehydrogenase
MEPM: Meropenem
PAPM/BP: Panipenem/Betamiprone
PSM: Propensity Score Matching
RBC: Red blood cell
SBT/ABPC: Sulbactam/Ampicillin
SBT/CPZ: Sulbactam/Cefoperazone
SCr: Serum creatinine
SMD: Standardized mean difference
TAZ/PIPC: Tazobactam/Piperacillin
WBC: White blood cell
WHO: World Health Organization
