# Supplementary material for: Impact of hospital formulary intervention on carbapenem use: a segmented time-series analysis of consumption and a propensity score-matched non-inferiority study of treatment efficacy
Source: J Pharm Health Care Sci. 2025 Jan 29;11:7. doi: 10.1186/s40780-025-00409-6 (PMC11776234; doi:10.1186/s40780-025-00409-6)
Supplement: Supplementary file 1 — Supplementary Material 1 [file 40780_2025_409_MOESM1_ESM.docx]

Appendix 1. Hospital Formulary Intervention on Carbapenem Usage at Yokohama City University Hospital

| **Recommendation** | **Medication** | **Generic** | **Price per vial ^*1^** | **Price per day ^*2^** |
| --- | --- | --- | --- | --- |
| 1^st^ | Meropenem (MEPM) | ○ | \ 581.0 | \ 3,486.0 |
| 2^nd^ | Doripenem (DRPM) |  | \ 1,517.0 | \ 4,551.0 |
|  | Imipenem/Cylastatin (IPM/CS) | ○ | \ 892.0 | \ 3,568.0 |

*1: Drug prices at October 2018.

*2: Standard daily dosage based on package insert

Appendix 2. Exclusion Criteria

| Exclusion Criteria |
| --- |
| Patients under 15 years of age at the start of treatment. |
| Patients with a history of treatment for the same condition at the start of the study period, or those currently undergoing treatment. |
| Patients who continued antibiotic treatment after the last day of the study period. |
| Patients with multiple treatment histories for the same condition during the study period, with treatment history in both periods. |
| Patients who underwent surgery within 12 hours for lower gastrointestinal perforation. |
| Patients who underwent surgery within 24 hours for a perforated gastroduodenal ulcer. |
| Patients who underwent open abdominal drainage. |
| Patients with confirmed abscess formation who did not receive appropriate drainage or other interventions. |
| Patients whose symptoms were already improving due to surgical interventions such as drainage before the use of antibiotics. |
| Patients with an APACHE II score greater than 15, indicating severe illness. |

Appendix 3. Distribution of Disease Groups and Treatment Outcomes Before and After the Intervention in the Matched Cohort

|  | Before | | After | |
| --- | --- | --- | --- | --- |
|  | n = 97 | | n = 97 | |
| Peritonitis, n (%) | 8 | (8.2) | 8 | (8.2) |
| Excellent | 1 |  | 2 |  |
| Effective | 6 |  | 5 |  |
| Ineffective | 1 |  | 1 |  |
| intra-abdominal abscess, n (%) | 23 | (23.7) | 26 | (26.8) |
| Excellent | 6 |  | 4 |  |
| Effective | 18 |  | 12 |  |
| Ineffective | 2 |  | 7 |  |
| hepatic/biliary tract infections, n (%) | 66 | (68.0) | 63 | (64.9) |
| Excellent | 6 |  | 6 |  |
| Effective | 47 |  | 48 |  |
| Ineffective | 10 |  | 12 |  |
